# Network-based drug sensitivity prediction

**DOI:** 10.1186/s12920-020-00829-3

**Published:** 2020-12-28

**Authors:** Khandakar Tanvir Ahmed, Sunho Park, Qibing Jiang, Yunku Yeu, TaeHyun Hwang, Wei Zhang

**Affiliations:** 1 Department of Computer Science, University of Central Florida, 4000 Central Florida Blvd, Orlando, FL 32816 USA; 2 Department of Quantitative Health Sciences, Lerner Research Institute, Cleveland Clinic, 9211 Euclid Ave, Cleveland, OH 44106 USA

**Keywords:** Drug sensitivity prediction, Gene co-expression network, Graph-based neural network, Network-based feature selection, Network embedding

## Abstract

**Background:**

Drug sensitivity prediction and drug responsive biomarker selection on high-throughput genomic data is a critical step in drug discovery. Many computational methods have been developed to serve this purpose including several deep neural network models. However, the modular relations among genomic features have been largely ignored in these methods. To overcome this limitation, the role of the gene co-expression network on drug sensitivity prediction is investigated in this study.

**Methods:**

In this paper, we first introduce a network-based method to identify representative features for drug response prediction by using the gene co-expression network. Then, two graph-based neural network models are proposed and both models integrate gene network information directly into neural network for outcome prediction. Next, we present a large-scale comparative study among the proposed network-based methods, canonical prediction algorithms (i.e., Elastic Net, Random Forest, Partial Least Squares Regression, and Support Vector Regression), and deep neural network models for drug sensitivity prediction. All the source code and processed datasets in this study are available at https://github.com/compbiolabucf/drug-sensitivity-prediction.

**Results:**

In the comparison of different feature selection methods and prediction methods on a non-small cell lung cancer (NSCLC) cell line RNA-seq gene expression dataset with 50 different drug treatments, we found that (1) the network-based feature selection method improves the prediction performance compared to Pearson correlation coefficients; (2) Random Forest outperforms all the other canonical prediction algorithms and deep neural network models; (3) the proposed graph-based neural network models show better prediction performance compared to deep neural network model; (4) the prediction performance is drug dependent and it may relate to the drug’s mechanism of action.

**Conclusions:**

Network-based feature selection method and prediction models improve the performance of the drug response prediction. The relations between the genomic features are more robust and stable compared to the correlation between each individual genomic feature and the drug response in high dimension and low sample size genomic datasets.

## Background

Powered by the high-throughput genomic technologies developed in the past two decades, personalized treatment has been enabled to understand complex diseases for individual patients. Diverse diseases such as cancer have gained increasing attention and a great number of works are going on to accelerate our understanding of the molecular basis of cancer through the application of genome analysis technologies [1–3]. However, due to the unavoidable patient heterogeneity, different patients have differential responses to the same treatment. Precision medicine takes the variabilities into account and allows clinicians to predict more accurately which treatment and prevention strategies for a particular cancer type will work on an individual patient.

However, the question of effective translation of high-throughput omics data from patient samples into prognosis and personalized treatment still remains. It needs a comprehensive study across many drugs, patients, diseases and profiling technologies, which is limited by time, expense and scope of the drugs that can be tested. Therefore, the researchers have been using omics data from tumor-derived cell lines and predictive algorithms as a substitute for the aforementioned study [4, 5]. The omics data includes but not limited to gene expression, mutation, and copy number variations.

Several studies have explored the use of state-of-the-art machine learning models, such as kernel-based methods [4, 6–8], Elastic Net [8, 9], nonlinear regression [10, 11], partial least-squares regression [4], and deep learning-based methods [12–17] to predict drug sensitivities. Most of the studies used omics data from CCLE (Cancer Cell Line Encyclopedia) [18] and GDSC (Genomics of Drug Sensitivity in Cancer) [19] to train the models and test the prediction power on an independent test set. However, different studies have found different models to be more accurate. Some studies [8, 20] concluded that Elastic Net performed better than other models whereas some other studies [4] found kernel-based methods to be better. Recently the deep neural network (DNN) based methods are becoming increasingly popular and several studies [12–15] have defined different models that used multi-omics data, often with drug structural information to predict drug sensitivity. Several other studies [16, 17] instead focused on drug synergy prediction. However, the modular relations among genomic features have been largely ignored in these studies.

It is well known that gene, transcript or protein isoforms do not function in isolation in the cell, but are integrated together as a network of interactions between cellular components. Cancer, as a complex disease, reflects the perturbations or breakdown of specific function modules in the complex cellular network, rather than a consequence of an abnormality in a single gene [21]. Thus, instead of considering the gene individually in the cancer studies, integrating network and high-throughput information together could probably improve the quality of the analysis [22]. Graph-based neural network recently has shown remarkable success in pattern recognition and data mining [23–25], and network-based embedding models are constructed by using random walk [24, 26] or neighborhood based method [27] to learn the network topological features. It is also proving its worth in the field of computational biology, such as drug-disease association prediction, drug-drug interaction prediction or protein-protein interaction prediction [28–33].

**Fig. 1:**
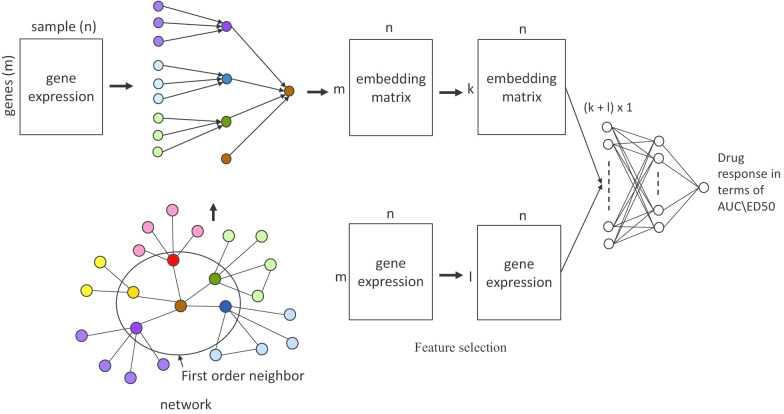
Workflow of the network-based embedding method. First, an embedding matrix is learned based on the local neighborhood structure in the gene co-expression network. Then, the features learned from the embedding matrix and the gene expression matrix are concatenated together to train a fully connected network for drug sensitivity prediction

**Fig. 2:**
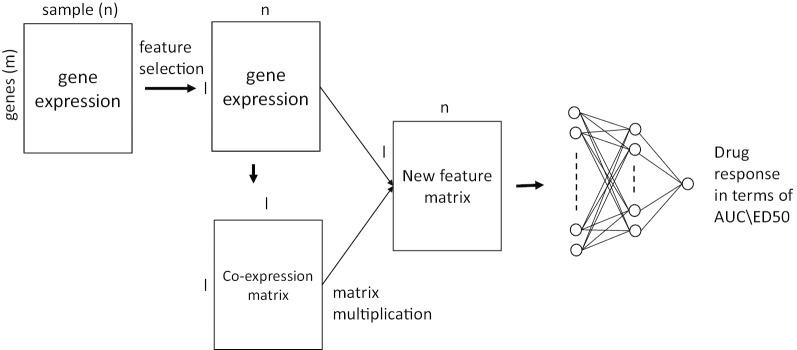
Workflow of the graphical neural network model. A multi-layer graphical neural network model is applied to learn the new feature representation of gene co-expression network and gene expression data. The outputs are applied to predict the drug response in terms of AUC and ED50

In this paper, we investigated the role of the gene co-expression network on drug sensitivity prediction. First, we compared a network-based feature selection method with the canonical feature selection method, and four different classification models were applied to the selected features to investigate the predictive power. Second, we look into two techniques, i.e., network-based embedding model (Fig. 1) and graphical neural network (GNN) model (Fig. 2), which integrate gene network information directly into a neural network for drug sensitivity prediction. A non-small cell lung cancer (NSCLC) cell line RNA-seq gene expression dataset with 50 different drug treatments was applied to evaluate the performance [34].

## Methods

In this section, we first introduce mathematical notations, and then a network-based learning model that is widely used for feature selection from a given data set [3]. Next, we discuss an advanced network-based embedding model to learn the representative features from the gene co-expression network and a graphical neural network model for drug sensitivity prediction. At the end of this section, we also introduce four canonical regression models and deep neural network as the baseline methods.

### Notations

In this paper, the gene expression data is denoted by $\varvec{X} =[{\varvec{x}}_1, {\varvec{x}}_2,\ldots ,{\varvec{x}}_m]$, where ${\varvec{x}}_i$ is the expression of the *i*-th gene across all the samples (i.e., cell lines). The dimension of the data set is $m\times n$, where *m* is the number of genes and *n* is the number of cell lines. The drug response information, i.e., area under the dose response curve (AUC) and median effective dose (ED50), is the target data set that measures the sensitivity of cell lines. It is denoted by $\varvec{y} =[{y}_1, {y}_2,\ldots ,{y}_n]$, and ${y}_j$ is the response of the *j*-th cell line to the drug. The drug sensitivity prediction can be defined as a regression problem. Drug response information of the test cell lines will be predicted based on the gene expression data and the known drug response information.

### Network-based feature selection model

We first introduce a network-based learning model that was applied successfully to identify molecular signatures in several variations [3, 35–37]. In the network, each vertex represents a gene and the edges represent the relations among the genes. Let $\varvec{A}\in {\mathbb {R}}^{m \times m}$ be the gene correlation matrix (i.e., the adjacency matrix of the gene co-expression network) based on the absolute value of the Pearson’s correlation coefficients between the pair of genes, where each $\varvec{A}_{ij}$ is the correlation between the two vectors, ${\varvec{x}}_i$ and ${\varvec{x}}_j$, which represent the *i*-th and the *j*-th genes. Then the features correlation matrix $\varvec{A}$ is used to construct a normalized graph Laplacian $\varvec{L} = \varvec{I}-\varvec{S}$, where $\varvec{S}=\varvec{D}^{-\frac{1}{2}}\varvec{A}\varvec{D}^{-\frac{1}{2}}$, $\varvec{D}$ is a diagonal matrix with the column-sum of $\varvec{A}$ on the diagonal entries, and $\varvec{I}$ is the identify matrix. Given a gene correlation matrix, the objective of the network-based learning model is to learn an assignment vector $\varvec{f}\in {\mathbb {R}}^{m\times 1}$, which represents the importance of each gene (i.e., vertex) for drug sensitivity prediction. The initial labeling is $\varvec{f}^{0}=\varvec{c}$, i.e., Pearson’s correlation coefficients between gene expression and the drug responses of the cell lines. The higher absolute value indicates the gene has more discriminative power. The network-based learning model assumes that the gene should be assigned similar importance scores if they are highly correlated in the network, which leads to the following objective function to be minimized:

1 $$\begin{aligned} {\mathcal {L}}(\varvec{f}) = \alpha \varvec{f}^{T}\varvec{L}\varvec{f}+(1-\alpha ) \left\| \varvec{f}-\varvec{c}\right\| _{2}^{2}, \end{aligned}$$

where $\alpha \in (0,1)$ is a parameter to balance the contributions of the two terms in Eq. (1), the first of which is the Laplacian term encouraging assigning similar importance scores to strongly connected vertices in the gene co-expression network; and the second term is the fitting term, which encourages consistency between the importance score and the initial score. The gene with high importance scores in $\varvec{f}$ will be selected for further analysis. The idea behind the network-based learning model is the relations between the genes are more robust and stable compared to the correlation between each individual gene and the drug response in high dimension and low sample size genomic datasets. In this study, the predictive power of the genes identified by the network-based learning model will be compared to the ones selected by Pearson correlation coefficients. Five different methods will be applied to evaluate the predictive power of the genes.

### Graph-based neural network models

Inspired by our network-based learning model and the recent advancements in deep representation learning for a network, we introduce two graph-based neural network models for drug sensitivity prediction in this subsection. In the first model, a network-based embedding method is proposed to learn the gene expression level of the target gene based on the local neighborhood structure. In the second model, a recently developed graphical neural network model is introduced by incorporating the gene co-expression network information.

#### Network-based embedding method

Different from encoding graph structure into low-dimensional embeddings [38], our proposed method is to learn a embedding matrix $\varvec{E} \in {\mathbb {R}}^{m \times n}$ that represents the expression level of the target node based on its local neighborhood structure as shown in Fig. 1, where each row in $\varvec{E}$ represents the local network information of its corresponding row in $\varvec{X}$. For each target node (gene) *v*, we constructed a partially connected shallow neural network (SNN) to encode the network information from its neighbors. We defined the top three correlated genes of gene *v* based on gene co-expression network and consider them as first order neighbors. Again the top three correlated genes with each of first order neighbors, in total nine genes were taken as second order neighbors. Both the first order neighbor and their direct second order neighbors were fed into the input of the SNN to learn the embedding information of the target node. The embedding vector of the node *v* is updated based on the following equation:

2 $$\begin{aligned} \varvec{e}_v^k=\sigma \left( \varvec{W}_k\sum _{u{\in }N(v)}\frac{\varvec{e}_u^{k-1}}{|N(v)|} +\varvec{e}_v^{k-1}\right) \quad for \quad k=1,2,3\ldots K, \end{aligned}$$

where *N*(*v*) denotes the first order and second order neighbors of the node *v* and $\varvec{e}_v^k$ is the embedding vector of the target node *v* in the *k-*th layer. $\sum _{u{\in }N(v)} \frac{\varvec{e}_u^{k-1}}{|N(v)|}$ is the average of neighbors’ embedding vectors from previous layer. $\varvec{W}_k$ is learnable weight parameters. $\sigma$($\cdot$) denotes the activation function. *K* is the number of layers. The initial embedding vector is $\varvec{e}_v^0 = \varvec{x}_v$. The loss function of the embeddings is defined as:

3 $$\begin{aligned} {\mathcal {L}}= \sum \limits _{(u,v) \in V \times V} \Vert \varvec{z}_u^T \varvec{z}_v - \varvec{A}_{u,v} \Vert ^2, \end{aligned}$$

where $\varvec{z}_v = \varvec{e}_v^K$ and *u* denotes the neighbor nodes of *v* and $\varvec{A}$ is the adjacency matrix of the gene co-expression network. In this loss function, we enforce that the relation between the learned embedding vectors should also be consistent with the original co-expression network. As shown in Fig. 1, the learned embedding vectors of the genes in the framework can be considered as a new set of features for drug sensitivity prediction. Once the embedding matrix is constructed, this matrix and gene expression matrix go through independent feature selection steps. We concatenated the selected features and fed that into a fully connected neural network to get a corresponding drug response as output. We used ReLU as activation function in the hidden layer and MSE as loss function.

#### Graphical neural network model

The network-based embedding method mentioned above only consider the local structures of the network to learn the representative features. In this subsection, we introduce a multi-layer graphical neural network model (GNN) which consider the global structure of the network that has been successfully applied in different domains [25, 28, 39]. Let $\tilde{\varvec{A}}$ denotes the adjacency matrix $\varvec{A}$ of the gene co-expression network plus the identity matrix and $\tilde{\varvec{D}}$ is a diagonal matrix with the column-sum of $\varvec{A}$ on the diagonal entries. A layer-wise propagation rule of the GNN can be defined as:

4 $$\begin{aligned} \varvec{H}^{k} = \sigma \left( \tilde{\varvec{D}}^{-\frac{1}{2}}\tilde{\varvec{A}} \tilde{\varvec{D}}^{-\frac{1}{2}}\varvec{H}^{k-1}\varvec{W}^{k-1}\right) , \end{aligned}$$

where $\varvec{H}^{k}$ is the output of *k-*th layer and $\varvec{H}^{0} =\varvec{X}$. $\varvec{W}^{k-1}$ is learnable weight parameters of the (*k*-1)th layer and $\sigma$($\cdot$) denotes the activation function. The output $\varvec{H}^{K}$ can be considered as new feature matrix for drug sensitivity prediction as shown in Fig. 2. In the framework, we used ReLU as activation function and MSE as loss function.

### Alternative methods for comparison and evaluation

For more insight in the drug sensitivity prediction problem by using gene expression data, we compared our proposed methods with four canonical prediction algorithms that were used in DREAM 7 - Drug Sensitivity Prediction Challenge [4]: Random Forest, Support Vector Regression (SVR), Elastic Net, and Partial Least Squares Regression (PLSR). In addition, the fully connected deep neural network (DNN) was also involved in the comparison in this study. These five prediction algorithms were applied to evaluate the discriminative power of the features identified by the network-based feature selection model in equation (1).

#### Random forest

Random Forest regression algorithm is a nonlinear multiple regression approach that performs bootstrap sampling of the training data to generate multitude of regression trees and outputs the mean prediction of individual trees [40]. All the trees in the forest run in parallel and find their results independently. In this baseline method, 500 trees were grown in the forest and 50 random features were selected for node splitting from all the features. This model was implemented via Python package sklearn.ensemble (RandomForestClassifier).

#### Elastic net

Elastic Net [41] is a regularized regression method to learn the coefficients ${\varvec{\beta }}$ following optimization problem:

$$\begin{aligned} \underset{{\varvec{\beta }}\in {{\mathbb {R}}}^{m}}{\min }R_{\lambda }{(\varvec{\beta })} =\underset{{\varvec{\beta }}\in {{\mathbb {R}}}^{m}}{\min }\left[ \frac{1}{2n} \sum _{i=1}^{n}{(y_{i}-x_{i}^{T}{\varvec{\beta }})}^{2}+{\lambda }P_{\alpha }({\varvec{\beta }})\right] , \end{aligned}$$

where

$$\begin{aligned} P_{\alpha }({\varvec{\beta }}) = \sum _{j=1}^{m}\left[ \frac{1}{2}(1-\alpha ){\beta }_{j}^{2} +\alpha |\beta _{j}|\right] . \end{aligned}$$

$P_{\alpha }$ is the elastic net penalty that linearly combines the $L_1$ and $L_2$ penalties of the coefficients. In our analysis, we fixed the $\alpha =0.5$ and the $\lambda$ was selected based on deviance likelihood ratio. This model was implemented via Python package sklearn.linear_model (ElasticNet).

#### Support vector regression

SVR is a kernel based method that can characterized by Vapnik-Chervonenkis control of the margin and the number of support vectors [42]. In our analysis, Radial Basis Function (RBF) kernel was used to train the model with the objective function

$$\begin{aligned} \underset{\alpha \ge 0}{\max }\sum _{i}^{n}{\alpha }_i -\frac{1}{2} \sum _{j,k}{\alpha }_j{\alpha }_k{y}_j{y}_k\exp (-\gamma \Vert {\varvec{x}}_{j} -{\varvec{x}}_{k}\Vert ^{2}), \end{aligned}$$

which subject to $0\le {\alpha }_i\le C$ for $\forall i$, and $\sum _{i}\alpha _{i}y_{i}=0$. In the analysis, *C* was fixed to 1, and $\gamma = 1/$(number of features in $\varvec{X}$*variance of $\varvec{X}$). This model was implemented via Python package sklearn.svm (SVR).

#### Partial least squares regression

PLSR is a statistical method that projects both independent variables (mRNA expression) and predicted variable (drug response values) in a new space and find a linear model between them. Specifically, PLSR is based on the basic latent component deposition to construct a matrix of latent component ${\varvec{T}}$ as a linear transformation of ${\varvec{X}}$: ${\varvec{T}} = {\varvec{XW}}$, where ${\varvec{T}}$ is a $n\times c$ matrix giving the *c* latent components for the *n* samples and ${\varvec{W}} = [{\varvec{w}}_1, {\varvec{w}}_2,\ldots ,{\varvec{w}}_c]$ is a $c\times m$ matrix of weights. The objective function need to be solved is:

$$\begin{aligned} {\underset{{{\varvec{w}}_i}\in {{\mathbb {R}}}^{m}}{\max }} {\varvec{w}}_i^{T} {\varvec{X}}^{T}{\varvec{yy}}^{T}{\varvec{X}}{\varvec{w}}_i, \end{aligned}$$

with $i=1,\ldots ,c$ and subject to ${\varvec{w}}_{i}^{T}{\varvec{w}}_{i}=1$ and ${\varvec{w}}_{i}^{T}{\varvec{X}}^{T}{\varvec{X}}{\varvec{w}}_{j}=0$, for $j=1,\ldots ,i-1$. More details on the PLSR method has been previously published in [43]. In the analysis, we fixed $c=1$. This model was implemented via Python package sklearn.cross_decomposition (PLSRegression).

#### Deep neural network

A two hidden layers fully-connected feedforward neural network model was also constructed for comparison. We used ReLU as activation on both hidden layers and Softmax on the output layer. Each neuron in the input layer represented the expression of one gene across all the cell lines. This model was implemented via pytorch [44].

## Results

In the experiments, we first compare the prediction power of the genes identified by network-based feature selection model and the genes identified by Pearson correlation coefficients. Four canonical prediction methods and DNN are applied to evaluate drug sensitivity prediction performance. Next, for the same selected features, we compare the different prediction methods (i.e., four canonical methods and DNN). The proposed graph-based neural network models are also involved in the comparison. The experiments are evaluated on 144 non-small cell lung cancer (NSCLC) cell lines screened by the same 50 drugs. Pearson correlation coefficients between predicted drug response (i.e., AUC and ED50) and the true response values are applied to estimate the prediction accuracy.

### NSCLC cell line dataset

The feature selection methods and prediction models were tested on 144 NSCLC cell lines RNA-seq gene expression dataset [34]. All the 144 cell lines were screened by the same drugs and the AUC and ED50 scores for each drug on each cell line are available in this study. Gene expression and drug response data went through significant pre-processing steps to make them compatible with each other. Firstly, the genes with low expression or low variance were filtered out. Secondly, if a gene has numerical value for more than 90% of the cell lines then we replaced all existing (if any) NaNs with the mean expression of that gene, otherwise, we filtered it out. Moreover, if a drug has the same response value from more than 80% of the cell lines, then we also filtered it out. In the end, we kept 50 drugs in this study.

**Table 1:** Drug response prediction results on the selected features

Feature selection method	Prediction methods				
	Elastic net	PLSR	Random forest	SVR	DNN
Correlation based	0.376 (0.041)	0.389 (0.033)	0.406 (0.026)	0.382 (0.037)	*0.346* (0.045)
Network based	0.392* (0.032)	*0.391* (0.033)	*0.421* (0.021)	*0.385* (0.035)	0.330 (0.057)

*The difference between the performances of the two feature selection methods is statistically significant (*p*-value $<\,0.01$). The better results between two methods are shown in italic

**Fig. 3:**
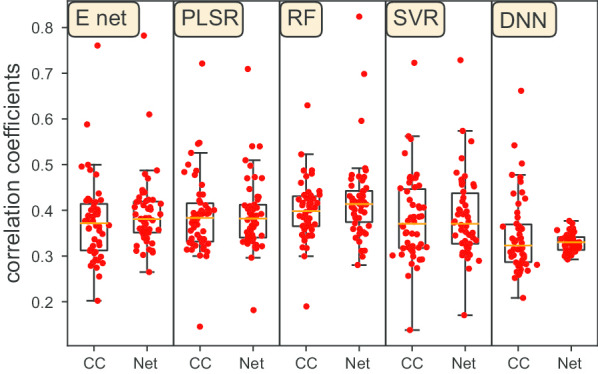
Drug response prediction results on the selected features. Each dot represents one drug. The mean correlation coefficients between the predicted AUC and the true AUC scores of 50 repeats for each drug are plotted

### Network-based feature selection methods improve prediction performance

To evaluate the quality of the genes identified by network-based feature selection method and Pearson correlation coefficients (i.e., the features are selected based the correlation coefficients values between drug response and the gene expression), we designed a drug sensitivity prediction task by the assumption that high quality of the identified molecular signatures can lead to better drug sensitivity prediction performance. In this task, the NSCLC cell line dataset was split into 70% as the training set, and 30% as the test set directly as the number of cell lines is limited. One hundred genes were selected in the training set by each feature selection method. For network-based feature selection method, the 100 genes were selected based on the top 100 importance scores. Whereas for correlation based approach, we select the genes with top 100 correlation coefficients. The drug sensitivity performance was measured on the test set. Five different prediction algorithms, Elastic Net (E net), PLSR, Random Forest (RF), SVM, and DNN were chosen to evaluate the results. We repeated the random splitting 50 times for each algorithm in each drug. To make the prediction results comparable among different feature selection methods and prediction algorithms, the same setup of training and test sets were used for all the methods in each splitting. Since no validation set was involved for model selection, the parameters in each prediction algorithm were fixed and the values are provided in the Methods section. The Pearson correlation coefficients between predicted drug response values and the true values (AUC) was applied to measure the performance.

The average Pearson correlation coefficients of the 2500 repeats (50 splittings for each drug and 50 drugs in total) for each prediction method are reported in Table 1 along with the *p*-value for each method inside the parentheses. As we can see from the table, prediction using all the methods except graph-based DNN produce statistically significant results (*p*-value < 0.05). The results also show that the genes selected by the network-based feature selection method perform better than the ones selected by Pearson correlation coefficients on four canonical prediction methods. In Fig. 3, we plot the prediction results. Each dot represents one drug in each prediction method. CC and Net denote Pearson correlation coefficients and network-based feature selection methods, respectively. Though CC performs better than the network-based feature selection method (Table 1) on DNN, the median value of the network-based method is higher than CC. It indicates that among the 50 drugs, the network-based method performs better on more cases than CC.

**Table 2:** Prediction results of the top-20 drugs

Drugs	E net CC	E net Net	PLSR CC	PLSR Net	RF CC	RF Net	SVR CC	SVR Net	DNN CC	DNN Net	GNN	Embed
SW157765	0.7606	0.7823	0.7212	0.7093	0.7956	*0.8239*	0.7228	0.7286	0.6615	0.3557	0.3406	0.3580
SW157692	0.5879	0.6098	0.5479	0.5402	0.6297	*0.6984*	0.5621	0.5738	0.5421	0.3354	0.3566	0.3364
SW134727	0.4957	0.4393	*0.5456*	0.5400	0.5226	0.4875	0.5248	0.5138	0.4722	0.3373	0.3654	0.3934
SW005017	0.4885	0.4793	0.5257	0.5097	0.4874	0.4690	*0.5562*	0.5510	0.5025	0.3769	0.3514	0.3393
SW072554	0.4784	0.4873	*0.5000*	0.4976	0.4870	0.4921	0.4505	0.4560	0.3644	0.3225	0.3821	0.3548
SW198886	*0.4997*	0.4444	0.4867	0.4702	0.4325	0.4074	0.4781	0.4673	0.4776	0.3072	0.4064	0.3553
SW197409	0.4163	0.4231	*0.4775*	0.4705	0.4401	0.4321	0.4709	0.4747	0.4392	0.3416	0.3853	0.3672
SW134963	0.4369	0.4034	0.4838	0.4727	*0.4744*	0.4193	0.4706	0.4616	0.4144	0.3462	0.3652	0.3691
SW006981	0.4223	0.4229	*0.4558*	0.4465	0.4162	0.3955	0.4541	0.4523	0.4367	0.3377	0.3439	0.3471
SW096640	0.3883	0.4111	0.4348	0.4292	*0.4803*	0.4774	0.4229	0.4196	0.3596	0.3413	0.3820	0.3806
SW148608	0.3968	0.4017	0.4167	0.4033	0.4110	0.4058	*0.4702*	0.4571	0.3950	0.3615	0.3637	0.3570
SW023297	0.3222	0.3711	0.3915	0.3846	0.4151	0.4313	0.4732	*0.4824*	0.4624	0.3605	0.3658	0.3341
SW074797	0.4224	*0.4696*	0.3923	0.4058	0.4223	0.4426	0.3768	0.3915	0.3614	0.3122	0.3955	0.3470
SW015134	0.4077	0.4283	0.3945	0.3924	0.4340	*0.4654*	0.3872	0.3909	0.3706	0.3374	0.3812	0.3459
SW043997	0.4006	0.3585	*0.4345*	0.4298	0.4160	0.4145	0.4062	0.4019	0.3655	0.3115	0.4248	0.3431
SW208072	0.3912	0.3910	0.4213	0.4236	0.4335	*0.4489*	0.4087	0.4109	0.3307	0.3247	0.3576	0.3394
SW113135	0.3942	0.4196	0.3974	0.4093	0.4603	*0.4727*	0.3643	0.3756	0.3152	0.3304	0.3820	0.3273
SW088073	0.2786	0.3024	0.4111	0.4020	0.4148	0.4146	*0.4607*	*0.4607*	0.4257	0.3127	0.3686	0.3536
SW018825	0.3759	0.3817	0.4028	0.4109	0.4093	0.4130	0.4118	*0.4278*	0.3383	0.3302	0.3661	0.3304
SW041995	0.3861	0.4071	0.3994	0.4125	0.4263	*0.4313*	0.3661	0.3681	0.3477	0.3405	0.3276	0.3554

The best results across all the methods are italic. Embed represents the network-based embedding method (Fig. 1) and GNN represents the graphical neural network model (Fig. 2)

### Canonical prediction methods perform better than DNN

Comparing the prediction performance among the four canonical prediction methods and DNN model, Random Forest has the best overall performance in Table 1 and Fig. 3. In addition, all the canonical prediction methods perform better than DNN. Due to the limited number of cell lines, DNN needs a larger sample size to train the model to get better performance. Moreover, the results in SVR show the largest variation while DNN on the genes selected by the network-based method shows the smallest variation.

In Table 2, we also report the prediction results on the top-20 drugs. The top-20 drugs were selected based on the performance across all the methods. In the top-20 drugs, the Random Forest on the features selected by the network-based method outperforms all the other methods (6 out of 20). Followed by PLSR on the features selected by the correlation coefficients (5 out of 20). DNN does not get the best performance on any of the drugs. We also observe that the responses of some drugs are easy to predict by any methods (e.g., SW157765 and SW157692), while some drugs are not (e.g., SW041995 in the Table 2). The prediction performance is drug dependent and it may relate to the drug’s mechanism of action (MOA). The available molecular structures of the top-20 drugs are listed in Additional file 1: Figure S1. Drug SW157765 accelerated metabolism and it is associated with activity in cells with high expression of the cytochrome p450 family member [34]. The selected gene signatures for SW15776 by our network-based feature selection model are enriched in several metabolic pathways (Additional file 1: Table S1).

**Table 3:** Drug response prediction performance

		Graph-based neural network	
	DNN CC	GNN	Network-based embedding
Correlation	0.3459 (0.045)	0.3742* (0.029)	0.3507* (0.042)

*The difference between the performance of graph-based neural network model and DNN is statistically significant (*p* value $<\,0.05$). The better results between two methods are shown in italic

**Fig. 4:**
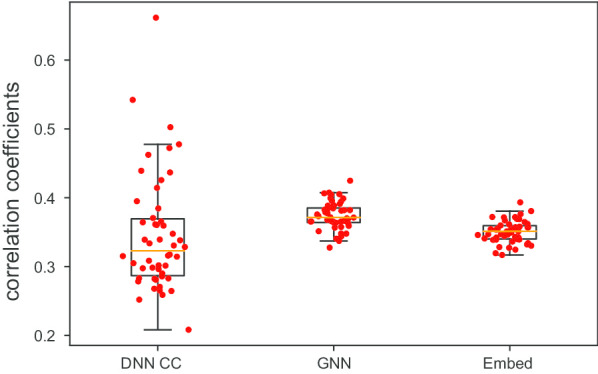
Drug response prediction results of the neural network based models. Each dot represents one drug. The mean correlation coefficients between the predicted AUC and the true AUC scores of 50 repeats for each drug are plotted. Embed represents the network-based embedding method (Fig. 1) and GNN represents the graphical neural network model (Fig. 2)

### Graph-based neural network models improve prediction performance compare to DNN

We introduced two graph-based neural network models, network-based embedding method and GNN in the Methods section, which integrate gene co-expression network into the drug sensitivity prediction. The performance of the two models was compared to the DNN and results are reported in Table 3 and Fig. 4. The training-test setups in this experiment were the same as the setups in the previous section. From the result, we can see that both graph-based neural network models improved the prediction performance compared to DNN which was not considering the gene network information in the modeling. GNN outperforms network-based embedding method and the performance of the top-20 drugs is also available in Table 2. Compared to the network-based embedding method, GNN considers the global structure of the gene co-expression network to learn the representative information for drug sensitivity prediction while the network-based embedding method only learns the representative features from the local neighborhood structure in the network. The network representative information learned by GNN may have more predictive power compared to the topological features learned from the local network structure by the embedding method.

Though the graph-based neural network models improve the performance of drug response prediction compared to DNN. The overall performance is still worse than the canonical prediction methods in Fig. 3 and Tables 1 and 2 since the neural network models suffer from overfitting and high-variance gradients in the high dimension and low sample size data. A larger sample size is needed to further improve the prediction performance.

### Running time

For a single iteration, correlation based feature selection takes 0.30 second of CPU time on average whereas network-based feature selection takes 1.16 second. Network-based feature selection method is always more time intensive than its correlation based counterpart. Time required for the predictive algorithm is insignificant compared to the feature selection step (both correlation based and network-based feature selection) except for random forest and DNN. The classifier function itself takes same amount of time for correlation based and network-based methods, for example SVR requires 0.0017 second for one prediction in both feature selection based models. Similarly, feature selection step takes same amount of time irrespective of the classifier used, for example correlation based feature selection always takes around 0.30 second. The codes were run using Intel(R) Core(TM) i7-8700 CPU @ 3.20GHz CPU.

## Discussion

Quantitative prediction of cellular responses to drugs is a challenging and valuable topic in personalized medicine. In the past decades, high-throughput technology has become a routine tool for monitoring genomic variations and it has been widely adopted for exploring drug response in the pharmaceutical research [45]. However, how to predict the effect of candidate therapeutic drugs and identify consistent molecular signatures using high-throughput technology is a challenging task due to heterogeneity of treatment effects, high dimension and low sample size, and statistical randomness or experimental noise in the data. Learning from the setup of the NCI DREAM challenge and the submitted drug sensitivity prediction algorithms [4], we did a comprehensive study on comparing the algorithms. In addition, a network-based feature selection method and two graph-based neural network models are introduced and involved in the comparison. These introduced methods fully explore modular co-expression structures along with gene discriminative power to provide more reliable representative features to improve the prediction performance. In general, network-based models can better capture the molecular interaction in the cellular system, which improves the predictive power of the selected genomic features. Network-based analysis also provides better consistency in genomic feature identification across different studies for the similar research purpose. We can conclude that network-based methods employ molecular and biomedical networks to extract useful genomic information, and build better predictive models for drug sensitivity prediction.

Currently the improvement for graph-based deep neural networks are limited in our study. To further increase the drug prediction accuracy, multi-omics data can be integrated together for the analysis. Multi-omics data capture genomic, epigenomic and transcriptomic characteristics of each cell line in the cohort and provide more accurate molecular signatures for drug response prediction on top of the large-scale biological features compared to single omics data only. TCGA, ICGC, and CCLE projects have profiled and analyzed large numbers of human tumor samples and cancer cell lines to measure the aberrations at the DNA, RNA, protein, and epigenetic levels. All these large-scale datasets can be integrated together for drug sensitivity prediction to overcome the overfitting problem in the deep neural network models. The integration of multi-omics data could make more biological information available for extraction e.g. genomic features from each modality, interaction of features within a modality, interaction of features across modalities. Our future study will extend this work to learn whether graph-based deep neural networks can achieve an edge over canonical methods while handling the complex interactive networks in multi-omics data. In addition, the chemical structural information for each drug can also be integrated together to further improve the performance.

## Conclusion

This study introduced a network-based feature selection method and two graph-based neural network models for drug sensitivity prediction. Comparing to the Pearson correlation coefficients for feature selection, four canonical prediction methods, and deep neural network on an NSCLC cell line dataset, we have made several useful observations. First, the network-based feature selection method identifies more representative features based on gene co-expression network for drug sensitivity prediction. Second, Random Forest outperforms all the other canonical prediction methods and deep neural network models, Third, the graph-based neural network models show better drug response prediction performance compared to DNN, however, it is still worse than the performance of the canonical prediction methods and a dataset with larger sample size is needed to further increase the prediction accuracy. Fourth, the prediction performance is drug dependent and it may relate to the drug’s mechanism of action (MOA). All the observations above were made based on the area under the dose response curve (AUC) values. Similar trends were also observed for ED50 values (results are not shown).

## Supplementary information

**Additional file 1:** Figure S1 and Table S1.

## Data Availability

The source code in this study is available at: https://github.com/compbiolabucf/Drug-sensitivity-prediction. The datasets used and analysed during the current study are available from the corresponding author on reasonable request.

## Abbreviations

NSCLC: Non-small cell lung cancer
CCLE: Cancer Cell Line Encyclopedia
DNN: Deep neural network
GNN: Graphical neural network
AUC: Area under the dose response curve
ED50: Median effective dose
SNN: Shallow neural network
MSE: Mean squared error
ReLU: Rectified linear units
E net: Elastic net
RF: Random forest
PLSR: Partial least squares regression
SVR: Support vector regression
CC: Pearson correlation coefficients
MOA: Mechanism of action
TCGA: The Cancer Genome Atlas
ICGC: International Cancer Genome Consortium
