# Supplementary material for: Network-based drug sensitivity prediction
Source: BMC Med Genomics. 2020 Dec 28;13(Suppl 11):193. doi: 10.1186/s12920-020-00829-3 (PMC7771088; doi:10.1186/s12920-020-00829-3)
Supplement: Supplementary file 1 — Additional file 1: Figure S1 and Table S1. [file 12920_2020_829_MOESM1_ESM.pdf]

Network-based Drug Sensitivity Prediction: supplemental document

1. MOLECULAR STRUCTURE OF DRUGS

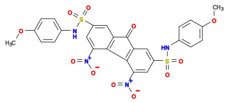

(a) SW005017

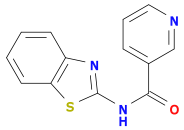

(b) SW006981

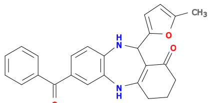

(c) SW015134

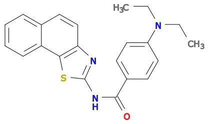

(d) SW018825

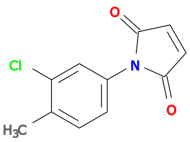

(e) SW023297

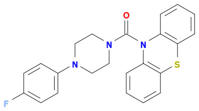

(f) SW041995

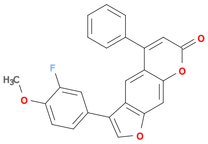

(g) SW043997

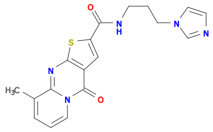

(h) SW072554

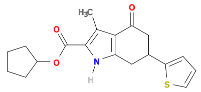

(i) SW074797

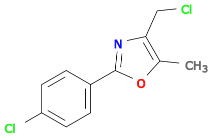

(j) SW088073

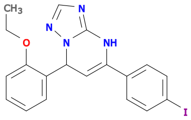

(k) SW096640

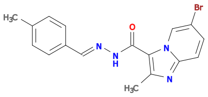

(l) SW113135

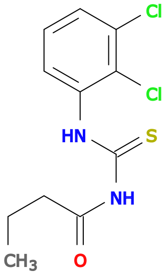

(m) SW134727

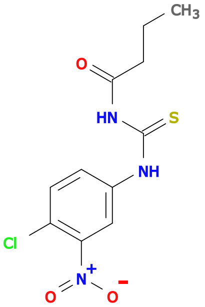

(n) SW134963

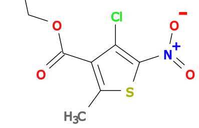

(o) SW148608

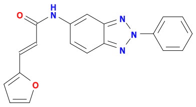

(p) SW157692

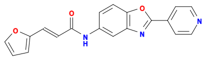

(q) SW157765

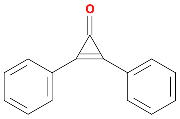

(r) SW197409

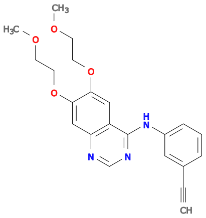

(s) SW198886

Fig. S1. Molecular structure of drugs

Figure S1 shows the molecular structures of our top predicted drugs from the list in Table 2 in manuscript.

2. SIGNIFICANCE OF SELECTED FEATURES

We performed an enrichment analysis on the 100 selected genes for our top predicted drug (SW157765) to show the biological importance of those genes. The result of the enrichment analysis is tabulated below.

Table S1. Enriched KEGG Pathways

KEGG Pathways	p-value
hsa00980:Metabolism of xenobiotics by cytochrome P450	2.87E-06
hsa00480:Glutathione metabolism	1.78E-05
hsa00140:Steroid hormone biosynthesis	7.82E-04
hsa05204:Chemical carcinogenesis	0.00198901
hsa00030:Pentose phosphate pathway	0.003762391
hsa00590:Arachidonic acid metabolism	0.015943241
hsa01100:Metabolic pathways	0.029288302
hsa01200:Carbon metabolism	0.049899021
hsa00051:Fructose and mannose metabolism	0.097635287
